# Cognitive workload across subjective and electrodermal measures in relation to previous-night sleep

**DOI:** 10.3389/fcogn.2026.1928964

**Published:** 2026-10-13

**Authors:** Maciej Lachowicz, Iwona Wilk, Igor Hrehorowicz, Szymon Bałamucki, Michał Lisowski, Dariusz Jamro, Grzegorz Zurek, Waldemar Andrzejewski

**Affiliations:** 1 Institute of Health Studies, DSW Ideis University, Wrocław, Poland; 2 Department of Fundamentals of Physical Therapy and Occupational Therapy, Wroclaw University of Health and Sport Sciences, Wrocław, Poland; 3 Department of Physical Education and Sport, General Tadeusz Kosciuszko Military University of Land Forces, Wrocław, Poland; 4 Department of Biological Principles of Physical Activity, Wroclaw University of Health and Sport Sciences, Wrocław, Poland

**Keywords:** cognitive effort, cognitive workload, Determination Test, electrodermal activity, galvanic skin response, NASA-TLX, psychophysiology, sleep duration

## Abstract

**Introduction:**

Cognitive workload is commonly assessed using both subjective and physiological indicators, although these measures may reflect different aspects of task-related strain. Sleep duration may further modify perceived task demand without producing proportional changes in physiological activation. This study examined the association between perceived cognitive workload and electrodermal task reactivity, and explored whether previous-night short sleep was associated with higher subjective workload without increased electrodermal activation.

**Methods:**

A total of 52 adults were initially assessed; two were excluded due to violation of predefined eligibility criteria, resulting in a final analytical sample of 50 participants. Participants reported previous-night sleep duration and subjective sleep quality, completed a 5-min resting baseline, and then performed the Determination Test from the Vienna Test System. Electrodermal activity was recorded continuously during baseline and task performance, while perceived workload was assessed after the task using the NASA Task Load Index. Task-related electrodermal reactivity was calculated as GSR task–baseline difference, and participants were classified into short-sleep (< 7 h) and normal-sleep (≥7 h) groups.

**Results:**

NASA-TLX total workload was not significantly associated with GSR task–baseline difference (rho = 0.126, *p* = 0.383). In the uncorrected threshold-based comparison, participants with short sleep reported higher perceived workload than those with normal sleep (64.44 ± 8.31 vs. 58.70 ± 9.57; *p* = 0.027), whereas GSR task–baseline difference was nearly identical between groups (4.57 ± 2.19 vs. 4.61 ± 2.93 μS; *p* = 0.884). However, the workload difference did not survive FDR correction across exploratory subgroup comparisons and should therefore be interpreted as a nominal finding.

**Discussion:**

Overall, perceived workload and electrodermal reactivity showed limited correspondence, whereas the sleep-related analyses did not provide consistent evidence of an association with either outcome.

## Introduction

Cognitive workload refers to the mental resources required to meet task demands under specific individual, task-related, and environmental conditions (Young et al., 2015). Rather than representing a single, directly observable state, it reflects a multidimensional construct involving mental demand, temporal demand, perceived performance, effort, frustration, and task-related strain (Hart and Staveland, 1988). For this reason, cognitive workload is usually inferred from subjective, behavioral, and psychophysiological indicators, as no single measure can fully capture the individual response to cognitive demand (Paas et al., 2003; Charles and Nixon, 2019; Tao et al., 2019; Ayres et al., 2021). Subjective measures are useful as they capture the participant's conscious appraisal of task difficulty, effort, time pressure, fatigue, and strain, which may not be fully reflected in task performance alone (Hart and Staveland, 1988; Galy et al., 2018). The NASA Task Load Index is one of the most established tools for assessing perceived workload as a multidimensional construct. Its dimensions, including mental demand, temporal demand, perceived performance, effort, and frustration, allow researchers to identify different subjective sources of task strain. Therefore, relying only on the total NASA-TLX score may obscure whether high workload reflects cognitive demand, compensatory effort, frustration, time pressure, or perceived poor performance (Galy et al., 2018).

Psychophysiological measures provide complementary information about bodily responses during task execution. Electrodermal activity reflects sympathetic nervous system activation and is sensitive to arousal during cognitive and emotional processing (Critchley, 2002; Boucsein, 2012). Experimental evidence shows that galvanic skin response can increase with cognitive load, supporting its use as an index of task-related arousal in controlled cognitive-task settings (Shi et al., 2007). However, EDA is not specific to cognitive effort, as it may also reflect stress, emotional salience, novelty, uncertainty, orienting responses, thermoregulation, movement artifacts, and individual autonomic differences (Critchley, 2002; Boucsein, 2012; Setz et al., 2010). For this reason, contemporary workload research favors multimodal assessment, combining subjective ratings with physiological markers such as EDA, heart rate, heart rate variability, respiration, eye-tracking, pupillometry, EEG, or fNIRS (Borghini et al., 2014; Charles and Nixon, 2019; Tao et al., 2019). No single physiological measure is universally valid across all tasks and populations, so subjective and psychophysiological indicators should be interpreted as complementary rather than interchangeable (Matthews et al., 2015; Ayres et al., 2021).

A further challenge is that subjective workload, task performance, and physiological activation do not necessarily change in parallel. Classic workload research shows that performance depends not only on task demands, but also on invested resources, resource efficiency, and competition between task components (Yeh and Wickens, 1988; Wickens, 2008). Thus, preserved performance during a demanding task may reflect compensatory effort rather than low workload (Hockey, 1997). Similarly, weak correspondence between NASA-TLX and GSR may indicate that a task was experienced as demanding without producing a proportional increase in mean electrodermal activation. Studies using EDA to estimate mental effort suggest that electrodermal features may be informative, yet their relationship with subjective effort varies depending on task context, signal features, and analytical strategy (Romine et al., 2022). One factor that may influence this subjective–physiological correspondence is sleep. In adults, sleep duration below 7 h is commonly treated as shorter than recommended sleep, as joint recommendations of the American Academy of Sleep Medicine and Sleep Research Society indicate that adults should sleep 7 or more h per night on a regular basis to promote optimal health (Watson et al., 2015). Similarly, the National Sleep Foundation recommends 7–9 h of sleep for young adults and adults (Hirshkowitz et al., 2015). Therefore, the < 7 h threshold used in the present study was selected as a pragmatic exploratory cut-off reflecting the lower boundary of recommended adult sleep duration.

Sleep-related effects on cognitive workload are unlikely to be limited to direct performance decline. Laboratory studies show that repeated restriction to 4–6 h of sleep produces cumulative, dose-dependent neurobehavioral impairment, while subjective sleepiness does not always track the progressive deterioration in performance (Van Dongen et al., 2003). Meta-analytic evidence also indicates that sleep deprivation most consistently affects attention-dependent functions, including vigilance, response stability, and processing speed, although effects vary across cognitive domains (Lim and Dinges, 2010). More broadly, sleep loss has been associated with increased sleepiness, fatigue, reduced alertness, mood changes, and poorer cognitive readiness, all of which may increase the perceived cost of task engagement even before clear performance failure occurs (Alhola and Polo-Kantola, 2007). Experimental evidence further suggests that cognitive workload and sleep restriction interact, increasing subjective fatigue and sleepiness under demanding task conditions (Goel et al., 2014). In partial sleep-deprivation protocols, subjective alertness, sustained attention, and subjective workload during working-memory tasks appear particularly sensitive to sleep loss (Lo et al., 2012). In aviation-related mental workload research, sleep restriction has also been associated with higher subjective workload during complex multitask performance (Pontiggia et al., 2024).

Mechanistically, insufficient sleep may increase perceived cognitive effort by increasing homeostatic sleep pressure and destabilizing wake-state regulation, consistent with two-process models of sleep regulation (Borbély, 1982; Borbély et al., 2016). With prolonged wakefulness or curtailed sleep, sleep-promoting pressure competes with arousal-promoting systems, producing attentional instability, response slowing, lapses, and greater intra-individual variability rather than a uniform decline in every cognitive domain. As a result, the participant may need to invest more top-down control to maintain task engagement and response accuracy. Neuroimaging and cognitive-control accounts suggest that performance after sleep loss can sometimes be partly preserved through compensatory recruitment of prefrontal and task-relevant control networks, but this compensation is likely to increase the subjective cost of task execution (Doran et al., 2001; Krause et al., 2017; Massar et al., 2019). This interpretation is especially relevant in healthy young adults, where subjective sleep quality does not always predict cognitive performance across working memory, executive function, or procedural learning tasks (Zavecz et al., 2020). The physiological expression of sleep-related effort is also context-dependent. Electrodermal activity may be sensitive to sleep deprivation, but this sensitivity depends on the analyzed signal feature and experimental setting. Higher-frequency or phasic EDA indices may detect sleep-deprivation effects more clearly than simple mean skin conductance level, and total sleep deprivation may amplify skin conductance responses to psychosocial stress (Posada-Quintero et al., 2017; Liu et al., 2015; Kurinec et al., 2022). However, these findings do not imply that naturalistic short sleep will necessarily produce higher mean GSR during every cognitive task. Sleep duration and sleep quality may therefore influence perceived cognitive effort through alertness instability, reduced readiness, and compensatory control rather than through a uniform increase in electrodermal activation.

Cognitive workload is also relevant to tasks in which cognitive processing is continuously coupled with motor responding. Perceptual–motor behavior involves linked processes of perception, response selection, and action execution, such that cognitive control can influence multiple stages between stimulus processing and goal-directed movement (Nieuwenhuys and Oudejans, 2012, 2017). Sensorimotor decision-making involves the selection, planning, and control of goal-directed movements based on incoming sensory information and task demands (Gallivan et al., 2018). Recent accounts of perceptual–motor coupling similarly describe perception and action as continuously interacting processes supported by sensorimotor integration rather than as independent stages (Du et al., 2026). These principles are relevant to cognitively demanding reaction tasks in which changing sensory information must be rapidly translated into appropriate motor responses. The Determination Test used in the present study has these characteristics: participants continuously discriminate visual and auditory stimuli, maintain stimulus–response mappings, select the corresponding response, and execute rapid manual responses while stimulus presentation rate adapts to individual performance. The task therefore allows subjective workload and autonomic activation to be assessed while cognitive processing is continuously coupled with motor responding.

### Research gap

Previous studies have examined subjective workload, physiological indicators of mental workload, sleep-related cognitive functioning, and cognitive–motor interference, but less is known about the correspondence between perceived workload and electrodermal reactivity during sustained cognition–movement performance. Physiological measures can discriminate changes in mental workload in many experimental settings, although their sensitivity varies across physiological measures and task domains (Charles and Nixon, 2019; Tao et al., 2019). This measurement problem is relevant to cognition–movement tasks as maintained behavioral performance does not necessarily imply that subjective effort and physiological activation change proportionally. The present study was designed to examine this potential subjective–electrodermal dissociation. Specifically, it assessed whether perceived cognitive workload corresponds with electrodermal task reactivity and whether sleep-related variables are associated more strongly with subjective workload than with physiological activation. The primary aim was to determine the relationship between perceived workload and electrodermal reactivity during a cognitive task. The secondary aim was to evaluate whether sleep duration and subjective sleep quality were associated with perceived workload and electrodermal activation. An additional exploratory aim was to examine whether short sleep was linked with greater subjective–physiological mismatch, defined as relatively higher perceived workload compared with electrodermal task reactivity. We hypothesized that perceived workload and electrodermal task reactivity would show weak correspondence. We also hypothesized that participants reporting less than 7 h of sleep would report higher perceived cognitive workload than participants sleeping 7 h or more, but would not show proportionally greater electrodermal task reactivity. Finally, we expected sleep-related variables to be more closely associated with perceived workload and subjective sleepiness than with electrodermal activation.

## Materials and methods

### Sample size justification and sensitivity analysis

No a priori sample size calculation was performed specifically for the present analysis. The dataset was derived from a broader psychophysiological protocol comprising two post-task recovery conditions: 15 min of classical back massage and 15 min of passive prone rest. Participants were allocated to the recovery condition before the analyzed measurements, but the assigned condition was administered only after completion of the baseline recording, Determination Test, and post-task NASA-TLX assessment. Thus, all variables examined in the present study were collected before the recovery manipulation. A total of 52 participants were initially assessed, and two were excluded before statistical analysis due to violation of predefined eligibility criteria, resulting in a final analytical sample of 50 participants with complete data. Participant flow and the study procedure are summarized in Figure 1. To contextualize the statistical sensitivity of the available sample, a sensitivity analysis was performed. For the primary correlation analysis between NASA-TLX total workload and GSR task–baseline difference, the minimum detectable correlation was estimated using Fisher's z transformation, assuming a two-tailed alpha level of 0.05 and statistical power of 80%. With *n* = 50, the available sample allowed detection of a correlation of approximately |rho| = 0.39. This indicates that the study was sensitive to moderate associations, whereas small associations may have remained undetected.

**Figure 1:**
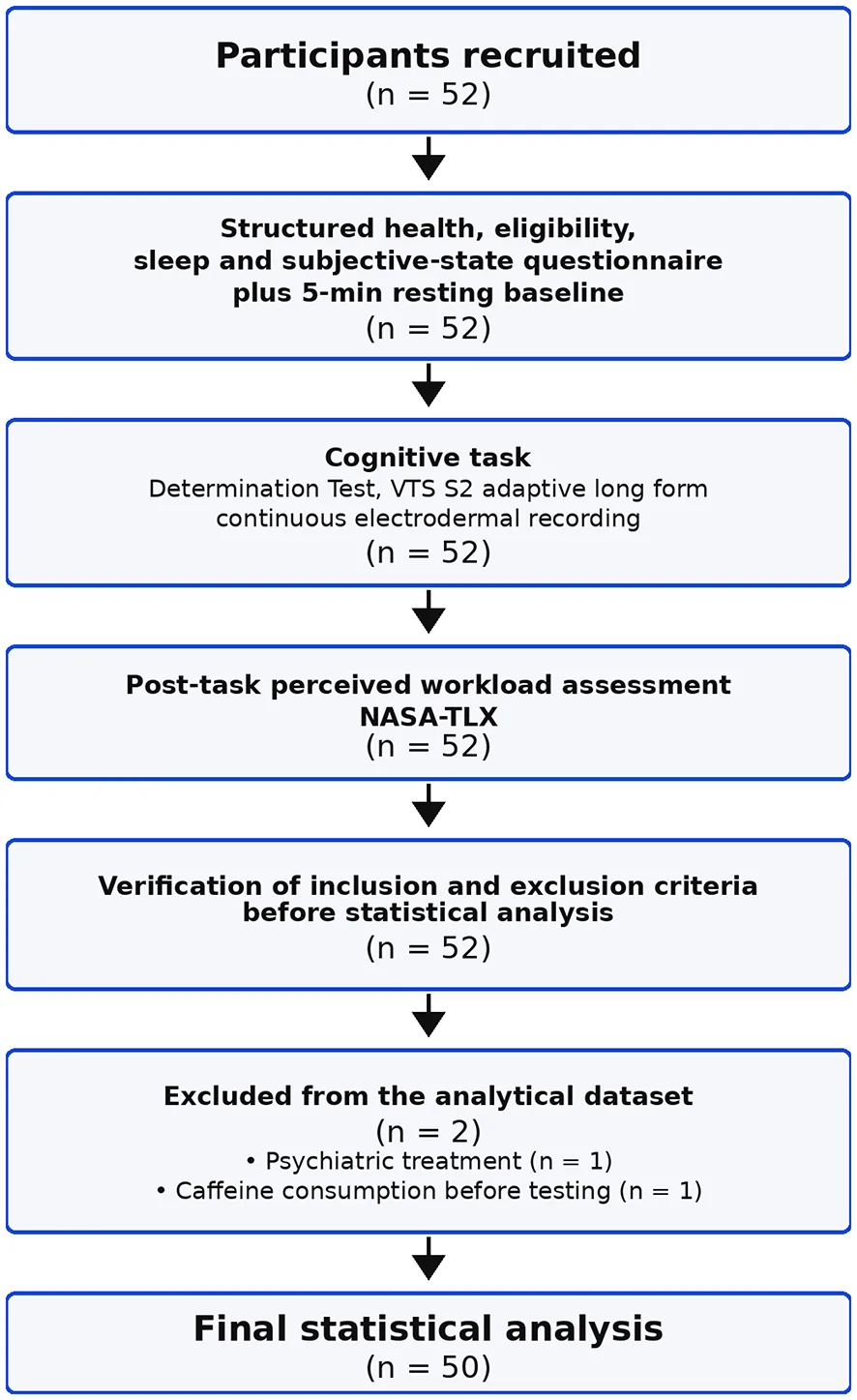
Flowchart of participant inclusion and analytical sample formation. The flowchart presents the study procedure from initial recruitment to final statistical analysis.

For the supplementary comparison between short-sleep and normal-sleep participants, the available group sizes were *n* = 27 and *n* = 23, respectively. Sensitivity for the between-group comparison was estimated using a two-sided independent-groups approximation, assuming alpha = 0.05 and power = 80%. With this allocation ratio, the available sample allowed detection of a large standardized between-group effect of approximately *d* = 0.80. This calculation was used only to describe the sensitivity of the available sample; the actual subgroup comparisons were conducted using the Mann–Whitney U test, with rank-biserial correlation reported as the effect-size measure. The sensitivity analysis was not used to reinterpret non-significant findings as evidence of absence of an effect. Instead, it was used to indicate the range of effects that the available sample was reasonably powered to detect. Therefore, non-significant findings, particularly those involving electrodermal reactivity and NASA–GSR mismatch indices, should be interpreted together with the observed effect sizes and the exploratory character of the analysis.

### Participants

The initial recruited sample consisted of 52 adults. All participants were right-handed and declared good general health. Before the experimental procedure, participants completed a structured health, eligibility, sleep, and subjective-state questionnaire. Formal eligibility verification for analytical inclusion was performed after completion of data collection and before statistical analysis. The screening included general health status, neurological and psychiatric history, other diagnosed diseases that could affect participation or physiological responses, medication or substance use affecting the central or autonomic nervous system, caffeine and nicotine intake in the 4 h preceding the study, intensive physical exercise in the 12 h preceding the study, and willingness to participate.

The inclusion criteria were age of at least 18 years, self-reported good general health, absence of contraindications to participation in the psychophysiological protocol, and provision of written informed consent. The exclusion criteria were neurological, psychiatric, or other diseases that could affect the course of the study or physiological responses; use of medication or substances influencing autonomic nervous system activity; lack of consent or withdrawal from the study; caffeine or nicotine consumption within 4 h before testing; and intensive physical exercise within 12 h before testing. Eligibility was verified based on participants' self-reported questionnaire responses.

After completion of data collection, the screening questionnaires were reviewed again before statistical analysis. Two participants were excluded from the analytical dataset due to violation of predefined eligibility criteria: one due to psychiatric treatment and one due to caffeine consumption before testing. Therefore, the final analytical sample consisted of 50 participants with complete sleep-related, perceived workload, cognitive-task, and electrodermal data.

All participants provided written informed consent before participation. The study adhered to the ethical standards outlined in the Declaration of Helsinki and its subsequent revisions. The study was approved by the Ethics Committee of Wroclaw University of Health and Sport Sciences; approval number: 46/2025, full approval available in Supplementary file 1.

### Cognitive task

Cognitive load was induced using the Determination Test from the Vienna Test System, version S2 adaptive form. The Determination Test is a computerized psychometric task designed to assess reactive stress tolerance, attention, and reaction speed under conditions of rapidly changing visual and auditory stimulation. According to the test description, the central measurement targets are reactive stress tolerance and reaction speed, while task execution requires discrimination of color and acoustic stimuli, maintenance of stimulus-response mappings, and rapid selection of appropriate responses according to predefined rules. The difficulty of the task results from the need to produce continuous, sustained, rapid, and varied reactions to changing stimuli (Lafayette Instrument Company, n.d.).

During the task, stimuli are presented continuously through visual and acoustic channels, and participants respond by pressing the corresponding buttons on the Vienna Test System response panel. The S2 form uses an adaptive presentation mode, in which stimulus presentation speed is adjusted to the respondent's performance level. Thus, the task maintains cognitive demand by adapting the rate of stimulation to individual performance rather than presenting all participants with a fixed, identical stimulus pace. Previous research using the adaptive Determination Test has characterized it as a measure of reactive stress tolerance and reaction speed under cognitively demanding conditions, with stimulus presentation adjusted to individual performance (Jongen et al., 2015; Jamro et al., 2026).

All participants completed the same standardized S2 adaptive long form under identical instructions. The task lasted approximately 8 min and ended according to the standard Vienna Test System procedure; no investigator-defined stopping rule or manual modification of the adaptive algorithm was applied. Because presentation rate was adapted to individual performance, stimulus rate and total stimulus exposure could vary across participants as an intended feature of the test. Performance was summarized using the global Vienna Test System outputs: median reaction time, numbers of correct, incorrect, and omitted responses, and accuracy. Trial-level presentation-rate trajectories and adaptive difficulty indices were not included in the exported dataset.

### Measurements

All measurements were conducted in the same room between 10:00 AM and 3:00 PM. This daytime window was selected to reduce boundary time-of-day effects on cognitive performance, fatigue, and sleepiness, since alertness, reaction speed, attention, and executive functioning may vary with time of day, time awake, and chronotype (Schmidt et al., 2007; Valdez, 2014; Vitale et al., 2023; Wiłkość-Debczyńska et al., 2023). Participants were seated during both the baseline and cognitive-task phases. Before the analytical baseline period, participants were allowed to settle in the testing position to support electrode stabilization and reduce immediate movement-related artifacts. Accelerometer and gyroscope channels were inspected during preprocessing to identify periods of excessive movement, abrupt repositioning, or procedural disturbance. Recordings were additionally checked for physiologically implausible values, abrupt signal discontinuities, and sensor-contact loss. Participants or phase segments with incomplete physiological data were excluded from the corresponding phase-based analysis. No interpolation was applied to reconstruct missing values within excluded or artifact-prone segments. No additional smoothing, filtering, or tonic/phasic decomposition was applied before calculating phase-level mean skin conductance. Environmental conditions were standardized procedurally by using the same testing room and daytime window for all participants. Room temperature and hand temperature were not recorded quantitatively; therefore, these factors were controlled procedurally but not included as measured covariates.

Before the cognitive task, participants completed a baseline questionnaire including demographic, anthropometric, sleep-related, and subjective state variables. Previous-night sleep duration was assessed by self-report and reported in hours. Subjective sleep quality was assessed using a numerical 0–10 scale, where higher values indicated better perceived sleep quality. No objective sleep assessment, such as actigraphy or polysomnography, was performed; therefore, these variables reflect perceived previous-night sleep rather than objectively measured sleep duration or sleep quality. Current physical fatigue, mental fatigue, stress, and pain/discomfort were also assessed using numerical 0–10 scales. Sleepiness was measured using the Karolinska Sleepiness Scale (Åkerstedt and Gillberg, 1990), ranging from 1 to 9, with higher scores indicating greater sleepiness.

Subjective workload after the cognitive task was assessed using the NASA Task Load Index. The NASA-TLX includes six dimensions: mental demand, physical demand, temporal demand, performance, effort, and frustration. Each dimension was scored on a 0–100 scale. In the performance dimension, lower scores indicated better perceived performance, with 0 representing perfect performance. The primary subjective workload outcome was the total average NASA-TLX score. Selected subscales, particularly mental demand, effort, frustration, and perceived performance, were analyzed as secondary subjective workload indicators.

Electrodermal activity was recorded continuously using a Shimmer3 GSR+ unit with ConsensysPRO software (Shimmer Sensing). The unit applies a 0.5-V bias across the GSR inputs and has a manufacturer-specified frequency range of DC−15.9 Hz. Data were acquired at an effective sampling frequency of approximately 128 Hz and expressed as skin conductance in microsiemens (μS). Two reusable dry electrodes supplied with the unit were attached to the volar surfaces of the proximal phalanges of the index and middle fingers of the non-dominant hand. No skin pretreatment was performed before electrode placement. Participants remained seated during a settling period before the 5-min analytical baseline. Electrodermal data were extracted from the 5-min resting baseline and the full cognitive-task period. Mean skin conductance was calculated separately for each period, and task-related electrodermal reactivity was expressed as the task–baseline difference in mean skin conductance:

**GSR task–baseline difference** **=** **mean task GSR – mean baseline GSR**.

To explore subjective–physiological mismatch, NASA-TLX total average and GSR task–baseline difference were standardized using z-scores. The NASA–GSR mismatch index was then calculated as:

**NASA–GSR Mismatch** **=** **z(NASA-TLX Total Average) – z(GSR task–baseline difference)**.

The recording and reporting procedures were described with reference to the SPR recommendations for electrodermal measurements and the manufacturer's documentation (Boucsein et al., 2012; Shimmer, 2018).

This index was used as an exploratory, sample-standardized indicator of relative discrepancy between perceived workload and electrodermal task reactivity. Since the index was mathematically derived from NASA-TLX total average and GSR task–baseline difference, associations between the mismatch index and its component variables were expected by construction and were not interpreted as independent validation of the index. Positive values indicated relatively higher perceived workload compared with electrodermal task reactivity. Negative values indicated relatively higher electrodermal task reactivity compared with perceived workload. The absolute value of this index was used as an additional exploratory indicator of the magnitude of subjective–physiological mismatch, regardless of direction.

## Statistical analysis

Statistical analyses were conducted using jamovi software (Version 2.6; The jamovi project, 2024). Descriptive statistics were calculated for demographic variables, sleep-related variables, subjective state variables, cognitive-task performance, NASA-TLX scores, electrodermal variables, and NASA–GSR mismatch indices. Continuous variables were described using means, standard deviations, medians, interquartile ranges, minimum and maximum values. Categorical variables were described using counts and percentages. The distribution of continuous variables was assessed using the Shapiro–Wilk test and visual inspection of histograms and Q–Q plots. As several variables showed non-normal distributions and some subjective measures were based on ordinal numerical scales, non-parametric analyses were used.

Participant demographic and baseline characteristics were additionally summarized for the total analytical sample and stratified according to previous-night sleep duration group (< 7 h vs. ≥7 h). Continuous demographic and baseline variables are presented as mean ± standard deviation. Categorical variables, including sex, are presented as counts. Between-group comparisons for continuous demographic and baseline variables were performed using the Mann–Whitney U test. Categorical variables were compared using Fisher's exact test. The comparison of previous-night sleep duration between groups was reported descriptively only, since sleep duration was used to define the short-sleep and normal-sleep groups. These demographic and baseline comparisons were used to characterize subgroup comparability and were not treated as primary hypothesis tests.

The primary analysis examined the association between perceived workload and electrodermal task reactivity. This relationship was assessed using Spearman's rank-order correlation coefficient between NASA-TLX total average and GSR task–baseline difference. Secondary correlation analyses examined associations between sleep duration, subjective sleep quality, Karolinska Sleepiness Scale score, NASA-TLX total average, selected NASA-TLX subscales, GSR task–baseline difference, and NASA–GSR mismatch indices. For the NASA-TLX performance dimension, lower scores indicated better perceived performance, with 0 representing perfect performance. Therefore, correlations involving this subscale were interpreted with this scoring direction in mind.

A threshold-based exploratory comparison was additionally conducted between participants reporting previous-night sleep duration below 7 h and those reporting 7 h or more. Between-group comparisons were performed using the Mann–Whitney U test. The compared outcomes included NASA-TLX total average, selected NASA-TLX subscales, GSR Baseline, GSR Task, GSR task–baseline difference, NASA–GSR Mismatch, NASA–GSR Absolute Mismatch, and cognitive-task performance indices.

For the exploratory short-sleep vs. normal-sleep subgroup comparisons, *p*-values were additionally adjusted using the Benjamini–Hochberg false discovery rate procedure (Benjamini and Hochberg, 1995). The correction was applied to the predefined family of 16 exploratory subgroup outcomes listed in Supplementary Table 1. Both uncorrected and FDR-adjusted *p*-values were reported.

Effect sizes were reported where appropriate. For Spearman correlations, rho values were interpreted as the strength and direction of associations. For Mann–Whitney U tests, rank-biserial correlation was used as the effect-size measure. The level of statistical significance was set at *p* < 0.05. Given the exploratory character of the analysis, exact *p*-values were reported and interpreted together with effect sizes and the direction of observed associations.

## Results

### Descriptive statistics

The final analytical sample consisted of 50 participants, including 27 women and 23 men. The mean age of the sample was 20.15 ± 1.07 years, with an age range of 18.54–23.46 years. Based on previous-night sleep duration, 27 participants were classified into the short-sleep group (< 7 h) and 23 into the normal-sleep group (≥7 h). Participant demographic and baseline characteristics stratified by previous-night sleep duration are presented in Table 1. No statistically significant differences between the short-sleep and normal-sleep groups were observed for age, sex distribution, height, body mass, BMI, Karolinska sleepiness score, physical fatigue, or mental fatigue. As expected, the groups differed in previous-night sleep duration and sleep quality.

**Table 1:** Participant demographic and baseline characteristics by previous-night sleep duration group.

Variable	Total sample	Short sleep < 7 h	Normal sleep ≥7 h	*p*
N	50	27	23	—
Sex, women/men (*n*)	27/23	12/15	15/8	0.166
Age (years)	20.15 ± 1.07	19.97 ± 0.74	20.35 ± 1.34	0.599
Height (cm)	174.28 ± 10.54	175.96 ± 11.00	172.30 ± 9.84	0.262
Body mass (kg)	67.52 ± 14.18	68.94 ± 16.29	65.77 ± 11.19	0.763
BMI (kg/m^2^)	21.96 ± 2.63	21.97 ± 2.92	21.95 ± 2.30	0.992
Previous-night sleep duration (h)	6.42 ± 1.55	5.30 ± 1.07	7.74 ± 0.81	< 0.001
Sleep quality (0–10)	6.38 ± 2.15	5.48 ± 2.05	7.43 ± 1.78	0.002
Karolinska sleepiness score (1–9)	4.54 ± 1.63	4.78 ± 1.65	4.26 ± 1.60	0.247
Physical fatigue (0–10)	4.20 ± 2.19	4.63 ± 2.02	3.70 ± 2.30	0.167
Mental fatigue (0–10)	4.52 ± 1.94	4.67 ± 1.90	4.35 ± 2.01	0.616

Descriptive statistics for the main sleep-related, subjective workload, electrodermal, mismatch, and cognitive-task variables are presented in Table 2 as means, standard deviations, medians, and interquartile ranges. Mean sleep duration during the previous night was 6.42 ± 1.55 h, with a median of 6.00 h [IQR: 6.00–7.75], and mean subjective sleep quality was 6.38 ± 2.15 points, with a median of 7.00 [IQR: 5.00–8.00]. The cognitive task produced a clear increase in electrodermal activity, with mean skin conductance increasing from 4.02 ± 2.75 μS during baseline to 8.61 ± 4.10 μS during the task. The corresponding median values were 3.60 μS [IQR: 2.13–5.04] for baseline and 7.44 μS [IQR: 5.48–11.46] for the task. The mean NASA-TLX total average score was 61.80 ± 9.28 points, with a median of 62.50 [IQR: 55.83–67.50], indicating moderate-to-high perceived workload after the cognitive task.

**Table 2:** Descriptive statistics for the main sleep-related, subjective workload, electrodermal, mismatch, and cognitive-task variables as mean ± standard deviation and median, given the non-normal distribution of several variables and the use of non-parametric analyses.

Variable	Mean ±SD	Median [IQR]
Sleep duration (h)	6.42 ± 1.55	6.00 [6.00–7.75]
Sleep quality (0–10)	6.38 ± 2.15	7.00 [5.00–8.00]
Karolinska sleepiness score (1–9)	4.54 ± 1.63	5.00 [3.00–6.00]
NASA-TLX total average	61.80 ± 9.28	62.50 [55.83–67.50]
NASA mental demand (0–100)	69.20 ± 15.10	75.00 [60.00–80.00]
NASA physical demand (0–100)	40.40 ± 22.15	42.50 [21.25–55.00]
NASA temporal demand (0–100)	77.80 ± 14.71	80.00 [70.00–90.00]
NASA performance (0–100)	52.10 ± 18.60	50.00 [36.25–65.00]
NASA effort (0–100)	70.40 ± 15.68	72.50 [65.00–80.00]
NASA frustration (0–100)	60.90 ± 24.76	65.00 [46.25–80.00]
GSR baseline (μS)	4.02 ± 2.75	3.60 [2.13–5.04]
GSR task (μS)	8.61 ± 4.10	7.44 [5.48–11.46]
GSR task–baseline difference (μS)	4.59 ± 2.53	3.92 [2.53–6.25]
NASA–GSR mismatch	0.00 ± 1.35	0.30 [−0.75–1.00]
NASA–GSR absolute mismatch	1.07 ± 0.81	0.92 [0.44–1.50]
DT median reaction time (s)	0.73 ± 0.05	0.73 [0.69–0.78]
DT correct responses	522.46 ± 44.50	523.00 [489.25–551.25]
DT incorrect responses	22.78 ± 13.71	21.00 [14.00–30.50]
DT omitted responses	30.48 ± 11.36	31.50 [23.25–38.00]
DT accuracy	0.91 ± 0.03	0.91 [0.89–0.93]

### Association between perceived workload and electrodermal task reactivity

The primary analysis showed no significant association between perceived workload and electrodermal task reactivity. NASA-TLX total average was not significantly correlated with GSR task–baseline difference (rho = 0.126, *p* = 0.383). This association is illustrated in Figure 2, and selected correlation coefficients are presented in Table 3. No statistically significant correlations with GSR task–baseline difference were observed for NASA mental demand (rho = 0.163, *p* = 0.258), NASA frustration (rho = −0.148, *p* = 0.304), or NASA performance (rho = 0.038, *p* = 0.796). NASA effort showed the largest correlation coefficient with GSR task–baseline difference (rho = 0.260), but the association was not statistically significant (*p* = 0.068). Within the sensitivity of the present sample, these analyses did not provide evidence that higher perceived workload was associated with greater electrodermal task reactivity.

**Figure 2:**
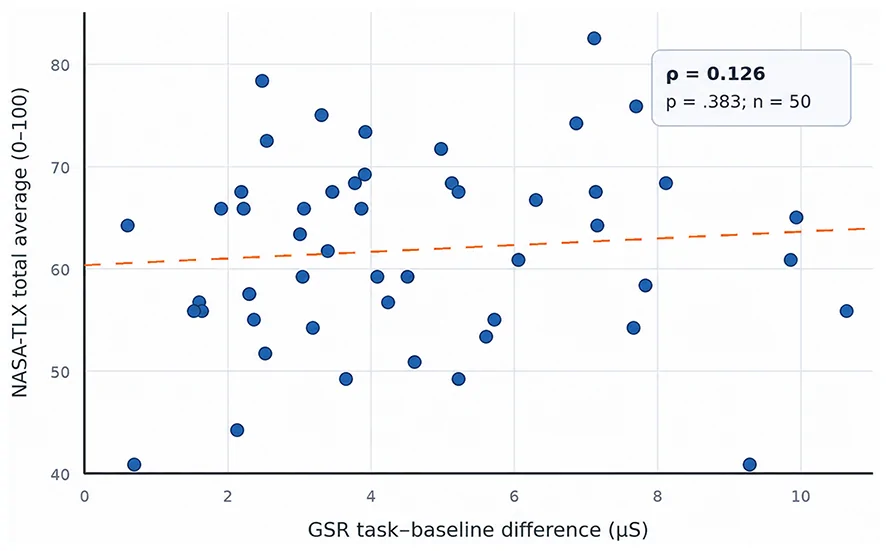
Association between perceived workload and electrodermal task reactivity. The scatterplot shows the association between the NASA-TLX total average score (0–100) and the task–baseline difference in mean skin conductance (μS). Each point represents one participant. The dashed least-squares line is included for visual orientation only; statistical inference was based on Spearman's rank-order correlation.

**Table 3:** Selected correlations among sleep variables, perceived workload, electrodermal task reactivity, and NASA-GSR mismatch.

Association	rho	*p*
NASA-TLX total average vs. GSR task–baseline difference	0.126	0.383
NASA effort vs. GSR task–baseline difference	0.260	0.068
Sleep duration vs. NASA-TLX total average	−0.223	0.120
Sleep duration vs. NASA performance	−0.263	0.065
Sleep duration vs. GSR task–baseline difference	−0.122	0.399
Sleep quality vs. NASA-TLX total average	−0.187	0.194
Sleep quality vs. GSR task–baseline difference	−0.087	0.547
Sleepiness vs. NASA-TLX total average	0.024	0.869
Sleepiness vs. GSR task–baseline difference	0.008	0.957
NASA–GSR mismatch vs. NASA-TLX total average	0.679	< 0.001
NASA–GSR mismatch vs. GSR task–baseline difference	−0.598	< 0.001

Values are Spearman rank-order correlation coefficients (rho) and corresponding *p*-values.

### Associations between sleep variables, perceived workload, and electrodermal activity

Sleep duration was not significantly associated with NASA-TLX total average (rho = −0.223, *p* = 0.120), NASA mental demand (rho = −0.169, *p* = 0.240), or NASA effort (rho = −0.193, *p* = 0.180). Sleep duration was not significantly associated with the NASA-TLX performance score (rho = −0.263, *p* = 0.065).

Sleep duration was not significantly associated with GSR task–baseline difference (rho = −0.122, p = 0.399). Similarly, subjective sleep quality was not significantly associated with NASA-TLX total average (rho = −0.187, *p* = 0.194) or GSR task–baseline difference (rho = −0.087, *p* = 0.547). Sleepiness measured with the Karolinska Sleepiness Scale was also not significantly associated with NASA-TLX total average (rho = 0.024, *p* = 0.869) or GSR task–baseline difference (rho = 0.008, *p* = 0.957).

Taken together, the continuous sleep-related analyses did not provide consistent evidence of a monotonic association between previous-night sleep duration and perceived workload. Therefore, the subsequent short-sleep vs. normal-sleep comparison was interpreted as a threshold-based exploratory analysis rather than as evidence of a linear sleep-duration effect.

### Previous-night short sleep vs. normal sleep

Participants were divided into a short-sleep group (< 7 h; *n* = 27) and a normal-sleep group (≥7 h; *n* = 23). Group comparisons are presented in Table 4, with selected outcomes illustrated in Figure 3. As expected, the short-sleep group reported significantly lower sleep quality than the normal-sleep group (5.48 ± 2.05 vs. 7.43 ± 1.78; *p* = 0.002).

**Table 4:** Short-sleep vs. normal-sleep group comparisons for subjective workload, electrodermal, mismatch, and cognitive-task outcomes.

Variable	Short sleep	Normal sleep	U	p	r_rb	95% CI for r_rb
Sleep quality	5.48 ± 2.05	7.43 ± 1.78	150.0	0.002	−0.517	[−0.755, −0.242]
Karolinska sleepiness	4.78 ± 1.65	4.26 ± 1.60	369.5	0.247	0.190	[−0.134, 0.499]
NASA-TLX total average	64.44 ± 8.31	58.70 ± 9.57	424.5	0.027	0.367	[0.055, 0.649]
NASA mental demand	72.78 ± 11.29	65.00 ± 17.97	390.0	0.121	0.256	[−0.071, 0.581]
NASA effort	72.96 ± 13.39	67.39 ± 17.83	373.5	0.220	0.203	[−0.124, 0.519]
NASA performance	54.44 ± 17.23	49.35 ± 20.13	369.5	0.252	0.190	[−0.129, 0.501]
GSR baseline (μS)	3.83 ± 2.10	4.25 ± 3.40	324.5	0.793	0.045	[−0.291, 0.385]
GSR task (μS)	8.40 ± 3.54	8.86 ± 4.75	319.5	0.869	0.029	[−0.312, 0.375]
GSR task–baseline difference (μS)	4.57 ± 2.19	4.61 ± 2.93	318.5	0.884	0.026	[−0.298, 0.359]
NASA–GSR mismatch	0.29 ± 1.03	−0.34 ± 1.60	385.0	0.150	0.240	[−0.101, 0.559]
Absolute mismatch	0.88 ± 0.60	1.30 ± 0.96	233.0	0.134	−0.250	[−0.556, 0.076]
DT median reaction time (s)	0.73 ± 0.06	0.74 ± 0.05	287.5	0.661	−0.074	[−0.385, 0.258]
DT accuracy	0.91 ± 0.04	0.91 ± 0.03	268.5	0.419	−0.135	[−0.459, 0.187]

Values are presented as mean ± standard deviation. Between-group comparisons were performed using the Mann–Whitney U test. Effect size is reported as rank-biserial correlation (r_rb) with 95% bootstrap confidence intervals. Positive r_rb values indicate higher values in the short-sleep group, whereas negative values indicate higher values in the normal-sleep group. For the NASA-TLX performance dimension, higher scores indicate worse perceived performance. GSR, galvanic skin response; DT, Determination Test; CI, confidence interval.

**Figure 3:**
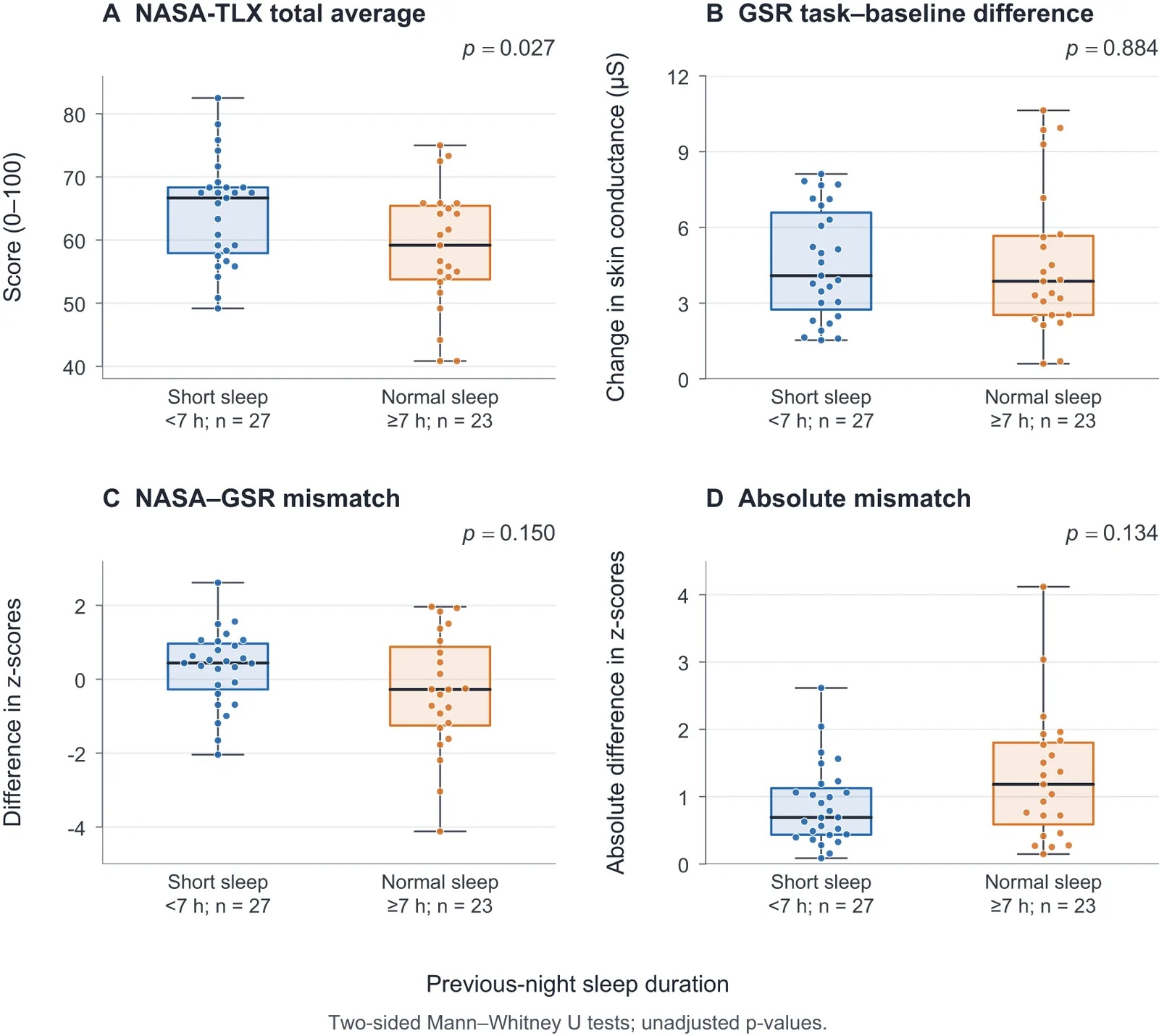
Short sleep vs. normal sleep across subjective and physiological outcomes. Box-and-jitter plots comparing NASA-TLX total average, GSR task–baseline difference, NASA–GSR mismatch, and absolute mismatch between participants sleeping less than 7 h and those sleeping 7 h or more. The *p*-values displayed in the panels are uncorrected Mann–Whitney U *p*-values. The NASA-TLX total average difference did not remain statistically significant after FDR correction (FDR-adjusted *p* = 0.216). Boxes indicate interquartile ranges, horizontal lines indicate medians, whiskers span the minimum and maximum values, and points represent individual participants.

The short-sleep group reported significantly higher perceived workload than the normal-sleep group. NASA-TLX total average was higher in participants sleeping less than 7 h (64.44 ± 8.31) than in participants sleeping 7 h or more (58.70 ± 9.57; *U* = 424.5, *p* = 0.027, rank-biserial *r* = 0.367). This group-based result should be interpreted as a threshold-based comparison rather than evidence of a linear dose-response relationship between sleep duration and perceived workload. No significant between-group difference was observed for GSR task–baseline difference (4.57 ± 2.19 vs. 4.61 ± 2.93 μS; *U* = 318.5, *p* = 0.884, rank-biserial *r* = 0.026).

These uncorrected subgroup findings were not interpreted as evidence of differential sleep effects across subjective and electrodermal outcomes and were considered together with the FDR-adjusted and direct mismatch analyses reported below.

To account for multiple exploratory short-sleep vs. normal-sleep comparisons, the Benjamini–Hochberg false discovery rate correction was applied. After correction, only sleep quality remained statistically significant (FDR-adjusted *p* = 0.032). The NASA-TLX total workload difference observed in the uncorrected analysis (*p* = 0.027) did not survive FDR correction (FDR-adjusted *p* = 0.216) and should therefore be regarded as nominal. The contrast between a nominal NASA-TLX difference and a non-significant GSR task–baseline difference does not establish that the sleep-group effects differed between the two outcomes. Consistently, neither NASA–GSR mismatch nor absolute mismatch differed significantly between groups. Thus, the subgroup analyses did not provide evidence of a sleep-related subjective–electrodermal dissociation.

### Exploratory NASA–GSR mismatch analysis

The NASA–GSR mismatch index was calculated to explore whether perceived workload was disproportionately high relative to electrodermal task reactivity. Positive values indicated relatively higher perceived workload compared with GSR reactivity, whereas negative values indicated relatively stronger GSR reactivity compared with perceived workload.

As expected from the construction of the index, NASA–GSR mismatch was positively associated with NASA-TLX total average (rho = 0.679, *p* < 0.001) and negatively associated with GSR task–baseline difference (rho = −0.598, *p* < 0.001). These associations reflect the mathematical definition of the index and are reported only to clarify its direction and behavior. They should not be interpreted as independent validation of the mismatch score. Sleep duration was not significantly associated with NASA–GSR mismatch (rho = −0.070, *p* = 0.630) or absolute mismatch (rho = 0.099, *p* = 0.493). Subjective sleep quality was also not significantly associated with NASA–GSR mismatch (rho = −0.140, *p* = 0.332). Group-based exploratory analysis showed that the short-sleep group had a mean NASA–GSR mismatch score of 0.29 ± 1.04 compared with −0.34 ± 1.60 in the normal-sleep group; however, this difference was not statistically significant (*p* = 0.150). Absolute mismatch also did not differ significantly between groups (*p* = 0.134).

## Discussion

The present study showed that perceived cognitive workload and electrodermal task reactivity did not closely correspond. Although the cognitive task produced a clear increase in skin conductance from baseline to task, the magnitude of this increase was not significantly associated with NASA-TLX total workload. This finding supports the view that cognitive workload should not be treated as a single, directly observable state, but rather as a multidimensional response expressed through subjective, behavioral, and physiological channels (Young et al., 2015). The weak association between perceived workload and GSR reactivity is consistent with the broader mental workload literature, which indicates that different workload indicators may diverge depending on task characteristics, individual strategy, and the type of measurement used (Wickens, 2008). Subjective workload reflects conscious appraisal of task demands, effort, frustration, and perceived coping capacity, whereas electrodermal activity reflects peripheral sympathetic arousal. Therefore, these measures can overlap, but they should not be interpreted as equivalent markers of the same process (Hart and Staveland, 1988). Marker-specific response patterns have also been observed across physiological systems following cognitive load, further indicating that individual physiological measures may capture different components of the task-related response (Lachowicz et al., 2026a,b).

The present task also places these findings within a cognition–movement setting. Determination Test performance requires continuous integration of sensory discrimination, response selection, and rapid manual motor execution, with stimulus presentation rate adapting to individual performance. Cognitive processing and motor responding are therefore functionally coupled throughout task execution. Research on cognitive–motor interference indicates that concurrent cognitive and motor demands can compete for processing resources and alter the neural resources required for task performance (Leone et al., 2017). In the present study, however, the objective was not to quantify cognitive–motor interference itself, but to examine whether subjective workload and electrodermal reactivity provided corresponding information while participants maintained performance in an adaptive sensorimotor task. The absence of a statistically significant NASA-TLX–GSR association indicates that these two measures did not show clear covariation in this setting, rather than demonstrating that either measure provides a superior index of cognition–movement performance.

This interpretation is also consistent with accounts suggesting that sleep loss may increase the subjective cost of effort and compensatory cognitive engagement even when performance impairment is not uniformly expressed (Engle-Friedman, 2014; Sullan et al., 2021). Peripheral physiological measures may detect workload-related changes, yet their validity depends on the physiological system recorded, the temporal resolution of analysis, and the type of cognitive demand imposed by the task (Charles and Nixon, 2019). In this context, the weak NASA-TLX–GSR association observed in the present study does not necessarily indicate measurement failure. Rather, it suggests that perceived workload and electrodermal reactivity captured partially distinct components of the cognitive-load response. Previous studies have shown that electrodermal activity may increase during cognitively demanding tasks and may be useful as an index of mental effort in controlled settings (Shi et al., 2007). However, EDA is not specific to cognitive workload. It reflects sympathetic nervous system activation and may be influenced by emotional arousal, attentional orienting, uncertainty, novelty, and individual autonomic reactivity (Critchley, 2002). Consequently, two participants may report similar subjective workload while showing different skin conductance responses, or may show similar electrodermal reactivity while experiencing different subjective levels of effort.

The sleep-related findings require a more tentative interpretation. Participants sleeping less than 7 h reported higher perceived workload than those sleeping 7 h or more, whereas mean skin conductance change from baseline to task was almost identical between groups. Previous experimental studies indicate that insufficient sleep can alter perceived workload during cognitively demanding performance, although the magnitude and expression of this effect depend on the sleep-loss protocol and task demands. Sleep deprivation has been associated with higher NASA-TLX workload despite preserved objective performance during simulated surgical tasks (Tomasko et al., 2012), while prolonged sleep deprivation during simulated driving has been associated with progressively higher subjective workload alongside deterioration in sustained-attention performance (Marando et al., 2022). Sleep restriction may also alter the willingness to invest cognitive effort, indicating that insufficient sleep can affect the subjective cost of cognitive engagement in addition to objective performance capacity (Jurgelis et al., 2022). Recent experimental evidence indicates that sleep restriction, perceived workload, fatigue, drowsiness, and behavioral performance can show distinguishable response patterns rather than changing proportionally (Rivas-Vidal et al., 2026). However, this group difference should be interpreted cautiously, as it was not consistently supported by the continuous sleep-related analyses. Sleep duration was not significantly correlated with NASA-TLX total workload when treated as a continuous variable. Similarly, subjective sleep quality and Karolinska sleepiness were not significantly associated with NASA-TLX total workload. Moreover, the group difference in NASA-TLX total workload did not survive FDR correction across exploratory short-sleep vs. normal-sleep comparisons. Therefore, the present findings do not provide consistent evidence of an association between previous-night sleep duration and perceived workload.

A more cautious interpretation is that the < 7 h cut-off may have captured a threshold-sensitive pattern in which participants reporting sleep below the recommended adult-duration boundary experienced the task as more demanding in the uncorrected group comparison. This pattern may reflect reduced readiness or greater subjective task cost in some participants, but it should not be interpreted as evidence that each additional hour of sleep is linearly associated with lower workload. The absence of significant associations involving sleep quality and sleepiness further indicates that the subjective workload difference cannot be explained simply by self-reported sleepiness. Thus, the present data are compatible with a compensatory-effort interpretation, but they do not directly demonstrate it. Broader sleep literature shows that sleep loss can affect alertness, vigilance, attention, and cognitive stability, with effects expressed differently across behavioral, subjective, and physiological domains (Lim and Dinges, 2010). It also aligns with evidence that chronic sleep restriction may produce cumulative neurobehavioral impairment even when individuals do not fully recognize the magnitude of their deficit (Van Dongen et al., 2003). In the present study, however, task performance did not clearly deteriorate in the short-sleep group. Therefore, if shorter sleep increased the experienced cost of task execution, this effect was expressed primarily as a nominal difference in perceived workload rather than as a consistent pattern across sleepiness, sleep quality, task performance, or mean electrodermal reactivity.

Compensatory-effort models provide one theoretical account relevant to cognitive-load assessment, as individuals may maintain performance under fatigue or stress through increased effort investment until compensatory capacity becomes insufficient (Hockey, 1997). Stable performance therefore does not necessarily imply low workload. The present findings also differ from controlled sleep-restriction studies in which sleep loss has affected both performance and physiological responses. Experimental sleep-restriction protocols typically involve controlled sleep curtailment, prolonged wakefulness, repeated testing, or additional stressors, which may produce stronger and more consistent effects than those observed under naturalistic variation in previous-night sleep (Banks and Dinges, 2007). Accordingly, the present results should not be treated as equivalent to effects observed in experimental sleep-restriction paradigms.

The exploratory NASA–GSR mismatch approach was intended to quantify the relative discrepancy between perceived workload and electrodermal task reactivity. The group difference in mismatch was not statistically significant and therefore did not provide evidence of a sleep-related difference in subjective–physiological mismatch. More generally, disagreement between subjective and physiological indicators may itself be informative rather than merely reflecting measurement error (Yeh and Wickens, 1988). Cognitive-load assessment should therefore not focus only on deciding which marker is “correct.” Subjective workload and GSR provide different information: NASA-TLX reflects the perceived demands of task performance, whereas GSR reflects electrodermal arousal during task execution. Multimodal approaches can therefore characterize whether cognitive demand is expressed subjectively, behaviorally, physiologically, or across combinations of these domains (Ayres et al., 2021; Lachowicz et al., 2026a).

This distinction may be relevant to applied activities that require sustained coupling between information processing and motor responding, including driving, aviation, military operations, competitive gaming, and other time-pressured human–machine interactions. Mental workload can affect human performance in complex human–machine systems, while the sensitivity of individual physiological measures varies across task conditions and measurement approaches (Charles and Nixon, 2019; Tao et al., 2019). In such settings, acceptable behavioral performance does not necessarily indicate low internal task demand, since performance may be maintained through continued allocation of cognitive resources (Hockey, 1997; Matthews et al., 2015). Consequently, behavioral performance, subjective workload, and physiological activation should not automatically be treated as interchangeable indicators. The present findings support this measurement distinction: NASA-TLX and mean electrodermal reactivity did not show statistically significant covariation despite both being assessed in relation to the same adaptive sensorimotor task. For cognition–movement research, multimodal assessment may therefore be useful for characterizing the conditions under which sensorimotor performance is maintained, although the present data do not establish whether divergence between subjective and physiological measures predicts subsequent performance deterioration.

## Limitations

Several limitations should be considered when interpreting the present findings.

Sleep was assessed using self-report and only in relation to the night preceding the experiment. Therefore, the study did not include objective sleep measurement, such as actigraphy or polysomnography, and did not capture habitual sleep patterns, accumulated sleep debt, chronotype, sleep regularity, or long-term sleep quality. In addition, the 7 h threshold used to distinguish short sleep from normal sleep is a simplified categorization and may not reflect individual sleep needs. The study used a naturalistic observational design rather than an experimentally controlled sleep-restriction protocol. Participants were not assigned to sleep conditions, and sleep duration was not manipulated. As a result, causal conclusions cannot be drawn, and the observed differences between short-sleep and normal-sleep participants may partly reflect unmeasured lifestyle, emotional, academic, or recovery-related factors.

Electrodermal activity was analyzed as mean skin conductance during baseline and during the cognitive task. This phase-level approach allowed task-related GSR reactivity to be estimated, but it did not distinguish tonic and phasic EDA components, such as skin conductance level, response frequency, response amplitude, or moment-to-moment changes during the task.

The sample size was based on the available analytical dataset rather than on an a priori power calculation for the present research question. Retrospective sensitivity analysis indicated that the study was powered mainly to detect moderate correlations and large between-group effects. Therefore, small associations or subtle subgroup differences may have remained undetected.

The NASA–GSR mismatch index was exploratory and sample-dependent, as it was calculated from z-standardized NASA-TLX total workload and GSR task–baseline difference values within the present sample. Its correlations with NASA-TLX and GSR task–baseline difference are expected by construction and do not constitute independent validation. Future studies should validate mismatch metrics against external criteria, such as performance breakdown, fatigue accumulation, repeated-task decline, or multimodal physiological markers.

## Future directions

Future studies should extend this approach by including a broader set of objective and semi-objective markers of cognitive load and fatigue. Electrodermal activity provides useful information about sympathetic arousal, but it does not capture the full complexity of cognitive strain. Therefore, future protocols should combine EDA with measures such as heart rate variability, pupillometry, eye-tracking, EEG, fNIRS, and detailed behavioral performance indices. Such multimodal assessment may help distinguish subjective fatigue, autonomic arousal, attentional effort, and actual performance breakdown more precisely.

Another important direction is to examine how commonly used countermeasures against sleep-related fatigue influence the relationship between perceived workload and physiological activation. Caffeine, energy drinks, and other stimulant-based strategies are frequently used to compensate for short sleep, especially by students, shift workers, drivers, and competitive gamers. Future research should determine whether these strategies reduce perceived workload, improve cognitive performance, increase physiological arousal, or merely mask subjective fatigue while leaving cognitive vulnerability present.

Finally, future studies should examine subjective–physiological correspondence across a broader range of cognitive and sensorimotor demands. This includes attention, vigilance, inhibitory control, working memory, decision-making, reaction time, and visuomotor coordination. Cognition–movement paradigms could further manipulate motor-response complexity, stimulus-response compatibility, response rate, or concurrent motor demands to determine whether increasing cognitive–motor demands alter the correspondence among subjective workload, physiological activation, and behavioral performance. Cognitive–motor interference research indicates that interactions between cognitive and motor demands depend on the resources required by the component tasks (Leone et al., 2017). Trial-level analyses may additionally determine whether changes in subjective or physiological workload precede response slowing, increased errors, or other deterioration in sensorimotor performance.

## Conclusions

The present study found limited correspondence between perceived cognitive workload and the task–baseline difference in mean skin conductance during an adaptive cognitive task. Participants reporting less than 7 h of sleep had higher NASA-TLX total workload in the uncorrected subgroup comparison, whereas mean electrodermal reactivity was nearly identical between groups. Because the workload difference did not survive FDR correction and the direct mismatch analyses were non-significant, this sleep-related pattern should be regarded as preliminary rather than confirmatory. Overall, the findings are consistent with subjective workload and electrodermal reactivity providing complementary, non-interchangeable information about task-related strain, while the possible influence of previous-night sleep requires further study. In adaptive tasks requiring continuous integration of cognitive processing with rapid motor responses, subjective workload and electrodermal activity may therefore provide complementary information about task-related demand. Future studies should determine whether divergence between these measures is associated with subsequent changes in reaction speed, response accuracy, or other indices of sensorimotor performance.

## Data Availability

The original contributions presented in the study are included in the article/Supplementary material, further inquiries can be directed to the corresponding author.
