# Supplementary material for: Cognitive workload across subjective and electrodermal measures in relation to previous-night sleep
Source: Front Cognit. 2026 Oct 13;5:1928964. doi: 10.3389/fcogn.2026.1928964 (PMC13709994; doi:10.3389/fcogn.2026.1928964)
Supplement: Supplementary file 1 [file Table_1.DOCX]

| **Variable** | **p raw** | **p FDR** |
| --- | --- | --- |
| Sleep quality | .002 | .032 |
| Karolinska sleepiness | .247 | .448 |
| NASA-TLX total average | .027 | .216 |
| NASA mental demand | .121 | .448 |
| NASA effort | .220 | .448 |
| NASA performance | .252 | .448 |
| GSR baseline | .793 | .884 |
| GSR task | .869 | .884 |
| GSR Task Delta | .884 | .884 |
| NASA-GSR mismatch | .150 | .448 |
| Absolute mismatch | .134 | .448 |
| DT median reaction time | .661 | .814 |
| DT accuracy | .419 | .610 |
| DT correct responses | .284 | .454 |
| DT incorrect responses | .495 | .660 |
| DT omitted responses | .231 | .448 |

Supplementary Table 1. False discovery rate correction for exploratory short-sleep versus normal-sleep comparisons.
